# Disease Activity and Progression of Disability in Multiple Sclerosis Patients Aged Over 50 With or Without Disease-Modifying Drug Treatment: A Retrospective Cohort Study

**DOI:** 10.7759/cureus.49927

**Published:** 2023-12-04

**Authors:** Akihiro Kondo, Ryotaro Ikeguchi, Kazuo Kitagawa, Yuko Shimizu

**Affiliations:** 1 Department of Neurology, Tokyo Women’s Medical University, Tokyo, JPN

**Keywords:** disease-modifying drug, multiple sclerosis, brainstem lesions, secondary progressive multiple sclerosis, elderly patients with multiple sclerosis, aging, disease activity

## Abstract

Background

This study aimed to clarify the need for disease-modifying drug (DMD) treatment in elderly patients with multiple sclerosis (MS) aged 50 years or older. MS is an autoimmune, demyelinating disease of the central nervous system that predominantly affects young women. Various DMDs are effective in preventing relapses and slowing the progression of disability in patients with MS. Although disease activity in MS is believed to decrease with aging, a consensus on the appropriate DMD treatment for elderly patients with MS is lacking.

Methodology

This study included elderly patients with MS (>50 years old). We compared the occurrence of relapses, worsening of disability, and conversion to secondary progressive MS (SPMS) between patients with DMD treatment and those without. Logistic regression analysis was performed to determine the predictors of these outcomes. Confounding factors were adjusted using propensity scores.

Results

From January 1991 to October 2022, 76 elderly patients with MS were included. The mean age at the last visit was 57.4 ± 6.3 years, with 51 patients being female. The mean age of onset of MS was 37.1 ± 10.1 years. Fifty-four patients were included in the DMD treatment group. The overall relapse rate was 38% (33% and 48% in the DMD treatment and untreated groups, respectively). No significant differences in relapse rates (p = 0.72) or in the Expanded Disability Status Scale (EDSS) scores were identified between the two groups. Kaplan-Meier curves showed no differences in the time to first relapse within five years between the two groups. Additionally, no significant predictors of relapse were identified. Among 61 patients with relapsing-remitting MS, 25% converted to SPMS during the observation period. Logistic regression analysis showed that older age at the final visit and the presence of brainstem lesions at the age of 50 years were associated with a higher rate of transition to SPMS.

Conclusions

In the present study, no significant difference was found in the rate of relapse, disability progression, and conversion to SPMS between the DMD treatment and untreated groups in elderly patients with MS. Therefore, in patients without long-term relapse, no poor prognostic functional factors or predictors of conversion to SPMS, discontinuation of DMDs may be considered. In addition, the presence of brainstem lesions at 50 years of age may predict the conversion to SPMS. Thus, the continuation of DMD or conversion to an appropriate DMD should be considered in patients with brainstem lesions at 50 years of age.

## Introduction

Multiple sclerosis (MS) is an autoimmune, inflammatory, demyelinating disease of the central nervous system that is particularly prevalent in young women. It is characterized by multiple lesions occurring in the central nervous system that disseminate in time and space. The associated neurological symptoms are various. Approximately half of the affected patients convert from relapsing-remitting MS (RRMS) to secondary progressive MS (SPMS), in which disability progresses regardless of relapse within 15-20 years of disease onset [1]. In Japan, disease-modifying drugs (DMDs) for MS have been covered by insurance since 2000 to prevent relapse. Seven types of DMDs, totaling eight medications, are currently available in Japan. Recently, the use of high-efficacy therapies, such as natalizumab (NTZ) or ofatumumab (OMA), at an early stage has been considered useful not only in the suppression of relapse but also in the suppression of disability progression. However, the usefulness of DMD treatment in elderly patients with MS, i.e., those over 50 years of age, remains unclear. The annual relapse rate decreases in patients with MS over 50 years of age compared to those under 50 years of age [2]. Additionally, gadolinium (Gd)-enhanced active lesions decrease with age [3]. Concerns also exist regarding increased medical expenses due to the long-term use of expensive DMDs and the increased risk of side effects due to aging [4]. In a previous study, no significant difference was identified in relapse rates between the DMD treatment group and the untreated group in patients with RRMS aged 50 years or older [5]. This study aimed to clarify the need for DMD in elderly patients by comparing the relapse, worsening of Expanded Disability Status Scale (EDSS) scores, and conversion from RRMS to SPMS between patients treated with DMD and those without DMD treatment, as well as examine the predictors for relapse, disability progression, and conversion to SPMS in elderly patients with MS.

## Materials and methods

**Figure 1:**
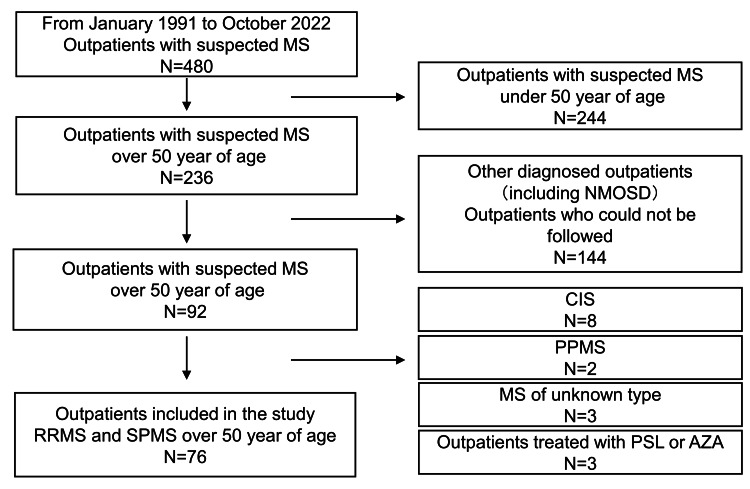
Flowchart of patient selection. Patients were followed up for at least three months and were diagnosed based on the McDonald criteria. AZA: azathioprine; CIS: clinically isolated syndrome; MS: multiple sclerosis; N: number of patients; NMOSD: neuromyelitis optica spectrum disorders; PSL: prednisolone; PPMS: primary progressive multiple sclerosis; RRMS: relapsing-remitting multiple sclerosis; SPMS: secondary progressive multiple sclerosis

**Figure 2:**
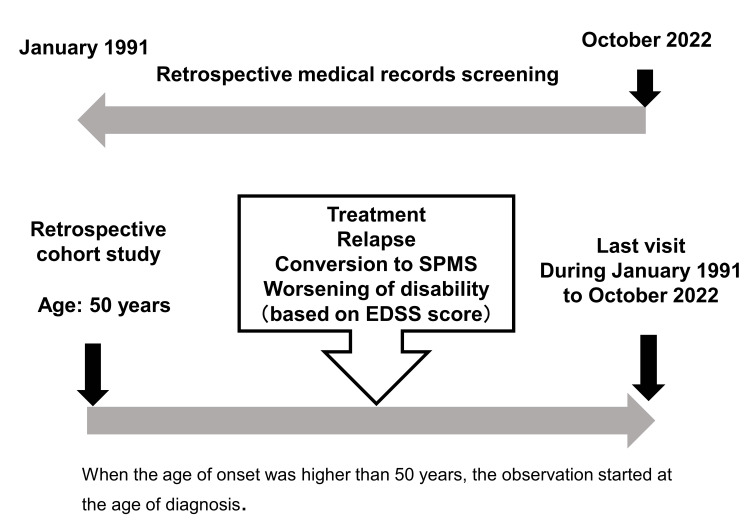
Research design. This was a retrospective cohort study. EDSS: Expanded Disability Status Scale; SPMS: secondary progressive multiple sclerosis

We conducted a retrospective study among patients with MS who had a medical history of outpatient visits to our clinic between January 1, 1991, and October 31, 2022. The patients included in this study met the 2017 McDonald diagnostic criteria for MS [6]. Patients with an unclear disease course, those who did not meet the 2017 McDonald criteria, and those with primary progressive MS were excluded from the study. In accordance with the established diagnostic criteria, neuromyelitis optica spectrum disorder was systematically ruled out in our study [7] (Figure 1). This study had a retrospective cohort design in which we examined DMD treatment, disease relapse, conversion to SPMS, and worsening of disability (EDSS score) in patients aged 50 years or older (Figure 2). While the exact age at which disease activity decreases in MS remains uncertain, prior studies have often used 50 years as a cutoff point, and a decrease in the incidence of contrast-enhancing MRI lesions has been observed [3,5,8]. In line with this precedent, our study also adopted the age of 50 years as the cutoff point for the investigation. Patients with late-onset MS, i.e., those who developed MS after 50 years of age, were observed from the age of diagnosis. The outcomes were relapse rate, worsening of disability, and conversion to SPMS in the DMD treatment and untreated groups. We also examined the predictors of relapse, worsening disability, and conversion to SPMS in the total, DMD treatment, and without DMD treatment groups. Regarding IgG index and OCB, cerebrospinal fluid tests were performed at the time of the initial diagnosis and during relapses, with the timing of measurement varying for each case. Furthermore, for IgG index values, when multiple tests are conducted, the higher value is adopted, and for OCB, if it is positive at least once, it is considered positive. Until 2005, high-resolution agarose gel electrophoresis was used to measure OCB. Measurements since then have been conducted using the isoelectric focusing method.

Statistical analysis

Categorical and continuous data were compared, respectively, using the chi-square test and Wilcoxon rank-sum test for non-parametric data. Statistical significance was defined as p-values <0.05. All statistical analyses were performed using JMP Pro 16.0.0 (512257SAS Institute Inc.).

Logistic regression models were developed to identify the DMD treatment-associated factors. We evaluated the relationship between DMD treatment and relapse, worsening of EDSS scores, and conversion to SPMS. Additionally, time-to-event analysis using the Kaplan-Meier method was performed to determine the onset of relapse.

Propensity score analysis

To account for the selection bias inherent to a retrospective study, a propensity score was calculated for each patient, which assessed DMD treatment for MS patients aged over 50 on observed covariates. The covariates used to build the propensity score were age at final visit, sex, age at disease onset, observation period, and disease subtype. All patients were stratified into quintiles based on their propensity scores and were included in the analyses. We assessed the adequacy of the propensity score specification by comparing the standardized differences in baseline covariates after stratification between the DMD and untreated groups. A standardized mean difference of <0.1 or p-values >0.05 was used to indicate no significant difference between the baseline covariates.

## Results

We examined 76 elderly patients with MS (age >50 years). Among them, 51 were in the DMD treatment group. The mean age at the last follow-up was 57.4 ± 6.3 years (mean ± SD), and 55 patients were females (72%, Table 1). The disease types included RRMS (n = 61) and SPMS (n = 15). The observation period (mean ± SD) was 7.4 ± 6.3 years. Twenty-four cases presented an EDSS score >3.0 at 50 years of age, while 52 cases had an EDSS score ≤3.0. A total of 51 patients underwent DMD treatment, including 22 cases on interferon β (IFN-β), one using glatiramer acetate (GA), 13 using dimethyl fumarate (DMF), 10 using fingolimod (FTY), two using NTZ, two using siponimod, and one using OMA. The age at the final visit was slightly lower in the DMD treatment group (55.4 ± 4.9 years) compared to the untreated group (61.4 ± 6.8 years, p = 0.00010). The frequency of SPMS was significantly higher in the DMD than in the untreated group (p = 0.00020). The observation period was significantly longer in the untreated (11.4 ± 6.8 years) than that in the DMD treatment group (5.5 ± 4.9 years, p = 0.00010). The IgG index was 0.84 ± 0.61 in the treatment group and 0.61 ± 0.27 in the untreated group, and there was a statistically significant difference with higher values in the treatment group (p = 0.0040). The frequency of spinal cord lesions at 50 years of age was higher in the DMD group than that in the untreated group (p = 0.0022). No differences were identified between the DMD treatment and untreated groups in terms of sex ratio, those with an EDSS score >3.0, OCB positivity, and the presence of cerebral and brainstem lesions on MRI at 50 years of age (Table 1).

**Table 1:** Demographic and clinical characteristics of patients with and without treatment. The p-value represents the relationship between the presence and absence of treatment. EDSS: Expanded Disability Status Scale; IFN-β: interferon-β; OCB: oligoclonal IgG bands; RRMS: relapsing-remitting multiple sclerosis; SD: standard deviation; SPMS: secondary progressive multiple sclerosis

	With treatment	Without treatment	Total	P-value
Number of patients	51	25	76	
Age of last visit, mean ± SD (years)	55.5 ± 4.9	61.4 ± 6.8	57.4 ± 6.3	0.00010
Sex				
Female	38	17	55	0.55
Male	13	8	21	
Age at disease onset, mean ± SD (years)	36.6 ± 9.4	38.1 ± 11.5	37.1 ± 10.1	0.46
Disease type				
RRMS	36	25	61	0.00020
SPMS	15	0	15	
Observation period, mean ± SD (years)	5.5 ± 4.9	11.4 ± 6.8	7.4 ± 6.3	0.00010
EDSS at 50 years of age				
EDSS >3.0	19 (40%)	5 (20%)	24 (30%)	0.12
EDSS ≦3.0	32 (60%)	20 (80%)	52 (70%)	
Type of reference treatment, at 50 years of age, n (%)				
IFN-β	22 (43%)		22 (29%)	
Dimethyl fumarate	13 (25%)		13 (17%)	
Fingolimod	10 (20%)		10 (13%)	
Natalizumab	2 (4.0%)		2 (3.0%)	
Siponimod	2 (4.0%)		2 (3.0%)	
Ofatumumab	1 (2.0%)		1 (1.3%)	
Glatiramer acetate	1 (2.0%)		1 (1.3%)	
Without treatment		25 (100%)	25 (33%)	
OCB (+)	26 (63%) (n = 41)	7 (39%) (n = 18)	33 (56%) (n = 59)	0.080
IgG index, mean ± SD	0.84 ± 0.61 (n = 42)	0.61 ± 0.27 (n = 23)	0.76 ± 0.41 (n = 65)	0.0040
Cerebral lesion at 50 years of age	41/44 (93%) (n = 44)	15/19 (79%) (n = 19)	56 (89%) (n = 63)	0.11
Brain stem lesion at 50 years of age	14/44 (32%) (n = 44)	2/19 (11%) (n = 19)	16 (25%) (n = 63)	0.060
Spinal cord lesion at 50 years of age	33/44 (75%) (n = 44)	6/18 (33%) (n = 18)	39 (63%) (n = 62)	0.0022

**Figure 3:**
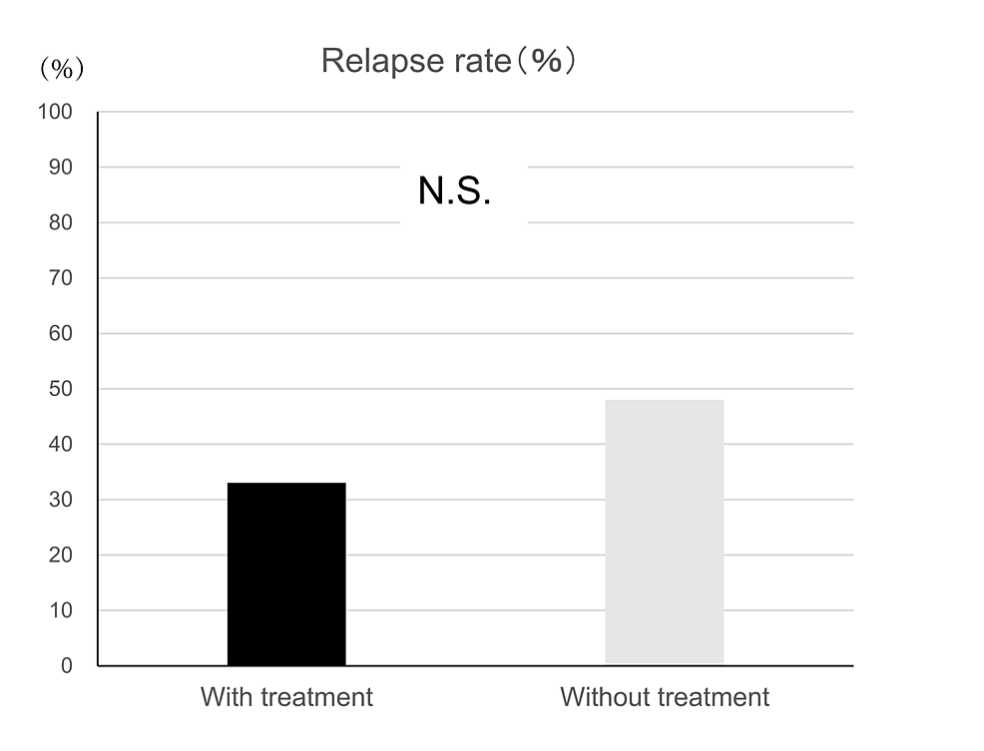
Relapse rates in elderly patients with MS. Relationship between treatment and relapse rate. MS: multiple sclerosis; N.S.: not significant

**Figure 4:**
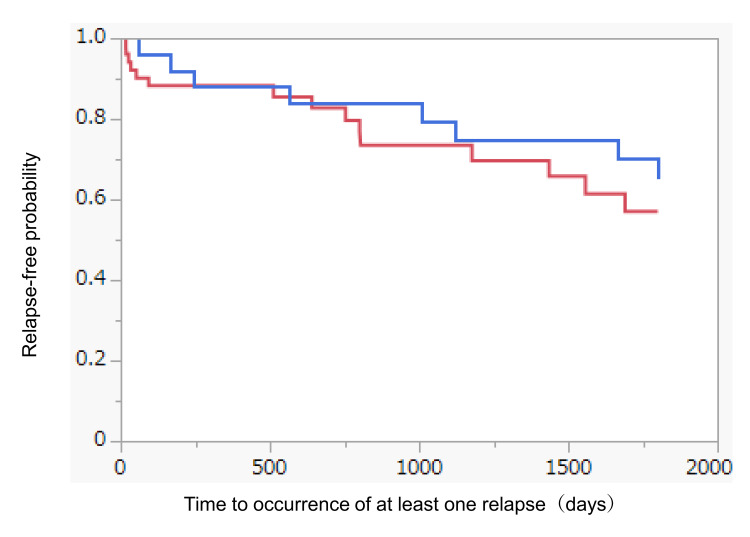
Relapse rates in elderly patients with MS. Kaplan-Meier curves for time to first relapse for the treatment and untreated groups, including patients aged 50 years or older. MS: multiple sclerosis

The relapse rates were 33% and 48% in the DMD treatment and untreated groups, respectively (Figure 3). The annualized relapse rates (ARRs) in each group were 0.12 and 0.38. Additionally, logistic regression analysis using propensity scores revealed no significant differences in the final age at the last visit, sex, disease subtype, and age of onset between the two groups (p = 0.72). The time to first relapse within a five-year period was compared between the DMD treatment and untreated groups using the Kaplan-Meier method. Although the DMD treatment group showed a slightly lower rate of relapse-free probability, no statistically significant difference was identified between the two groups (Figure 4).

**Figure 5:**
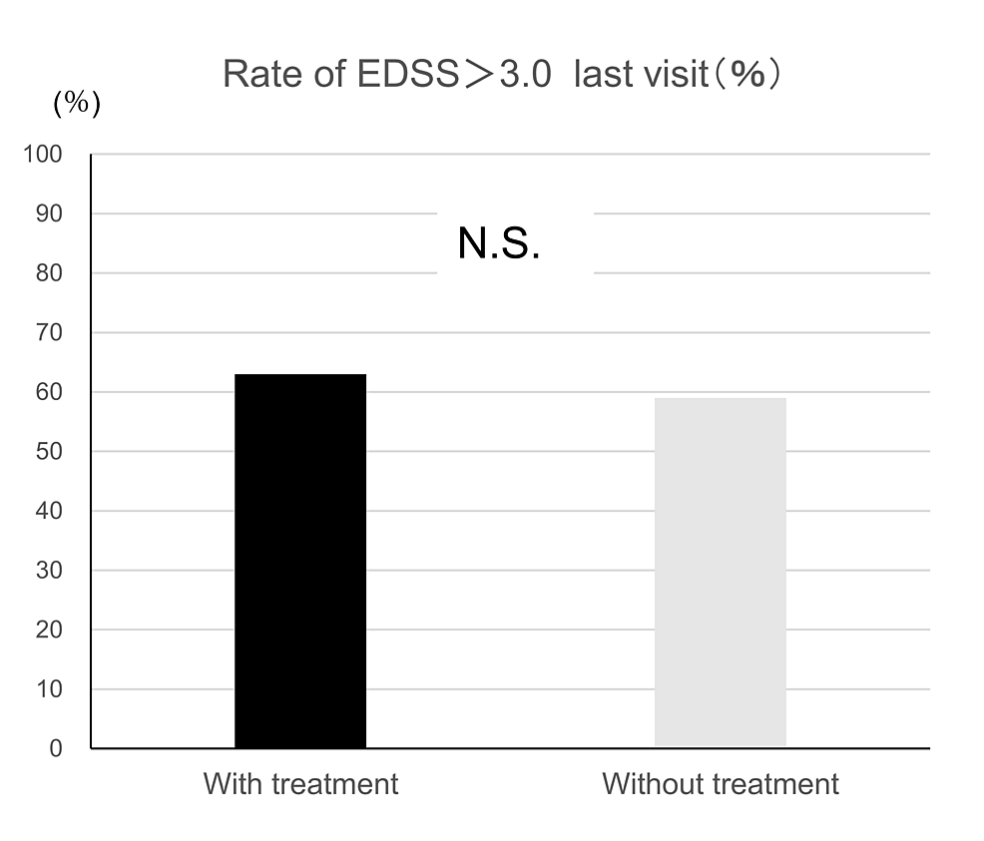
Relationship between treatment and EDSS scores at the last visit. EDSS: Expanded Disability Status Scale; N.S.: not significant

We compared the EDSS scores at the last follow-up between the DMD and untreated groups. In the DMD treatment group, 63% of the patients had an EDSS score >3, whereas those in the untreated group were 59% (p = 0.74). Additionally, we performed logistic regression analysis using propensity scores based on age at the last follow-up, sex, disease subtype, and observation period; no significant difference was found (p = 0.89) (Figure 5).

Although we compared the clinical characteristics, including the age at last visit, sex, age of onset, observation period, rate of EDSS scores >3.0 at 50 years of age, IgG index, and presence of central nervous system lesions (cerebral, brainstem, and spinal lesions) at 50 years of age between patients with and without relapse, no statistically significant difference was identified between the groups. In the patients who presented relapse, the DMD treatment consisted of IFN-β in nine cases, GA in one, DMF in four, FTY in two, and natalizumab in one. In contrast, among patients without relapse, 13 were treated with IFN-β, nine with DMF, eight with FTY, two with siponimod, one with NTZ, and one with OMA.

**Figure 6:**
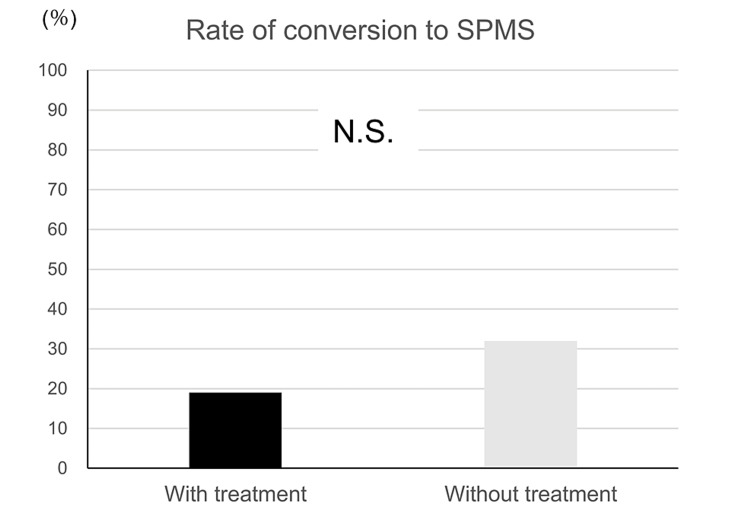
Rate of conversion to SPMS in patients aged over 50 years. N.S.: not significant; SPMS: secondary progressive multiple sclerosis

We investigated the conversion to SPMS in patients with RRMS aged 50 years in the DMD treatment group and untreated groups (Table 2). The DMD treatment and untreated groups included 15 and 46 patients, respectively. The final age at the last visit was 62.2 ± 6.7 years and 57.1 ± 6.0 years, respectively (p = 0.0067). The conversion rate to SPMS was 19% in the DMD treatment group and 32% in the untreated group (Figure 6). Logistic regression analysis using propensity scores did not reveal a statistically significant difference in conversion to SPMS between the DMD treatment and untreated groups (p = 0.50). Logistic regression analysis was performed to identify predictors of conversion from RRMS to SPMS; statistically significant differences were found in the final age at the last visit and in the presence of brainstem lesions at 50 years of age (odds ratios of 1.49 and 29.60, respectively, Table 3).

**Table 2:** Demographic and clinical characteristics of patients with and without conversion to SPMS. EDSS: Expanded Disability Status Scale; IFN-β: interferon-β; OCB: oligoclonal IgG bands; SD: standard deviation; SPMS: secondary progressive multiple sclerosis

	With conversion	Without conversion	P-value
Number of patients	15	46	
Age of last visit, mean ± SD (years)	62.2 ± 6.7	57.1 ± 6.0	0.0067
Sex			
Female	13	31	0.13
Male	2	15	
Age at disease onset, mean ± SD (years)	39.9 ± 12.4	37.6 ± 9.9	0.46
Observation period, mean ± SD (years)	8.4 ± 7.2	6.6 ± 5.5	0.48
EDSS at 50 years of age			
EDSS >3.0	6 (40%)	6 (13%)	0.031
EDSS ≦3.0	9 (60%)	40 (87%)	
Type of reference treatment at 50 years of age, n (%)			
Dimethyl fumarate	2 (13%)	9 (20%)	
Fingolimod	0 (0%)	4 (9.0%)	
Glatiramer acetate	0 (0%)	0 (0%)	
IFN-β	5 (33%)	16 (35%)	
Natalizumab	0 (0%)	0 (0%)	
Ofatumumab	0 (0%)	0 (0%)	
Without treatment	8 (53%)	17 (37%)	
OCB (+)	6 (50%) (n = 12)	19 (53%) (n = 36)	0.87
IgG index, mean ± SD	0.74 ± 0.33 (n = 14)	0.67 ± 0.39 (n = 41)	0.28
Cerebral lesion at 50 years of age	10 (100%) (n = 10)	32 (60%) (n = 38)	0.080
Brain stem lesion at 50 years of age	5 (50%) (n = 10)	4 (11%) (n = 38)	0.0087
Spinal cord lesion at 50 years of age	5 (56%) (n = 9)	20 (53%) (n = 38)	0.87
Relapse (+)	8 (53%) (n = 15)	16 (35%) (n = 46)	0.21

**Table 3:** Multivariate Cox model for the outcome “conversion to SPMS.” CI: confidence interval; SPMS: secondary progressive multiple sclerosis

	Odds ratio	95% CI	P-value
Sex	2.54	0.31-20.51	0.38
Age at last visit	1.49	1.05-2.13	0.03
Age of onset	0.91	0.79-1.05	0.20
Brainstem lesion at 50 years of age	29.60	1.77-494.64	0.02

## Discussion

This study aimed to clarify the need for DMD treatment in elderly patients with MS aged 50 years or older. Therefore, our study compared disease activity and conversion to SPMS in elderly patients with MS between the DMD treatment and untreated groups. Generally, disease activity, such as relapse and the presence of new or Gd-enhanced lesions on MRI, decreases in elderly patients with MS. According to a previous study, the ARR of MS decreases with age; for individuals in their 20s and 30s, the ARR is 0.26 in females and 0.32 in males, whereas for those in their 50s, it decreases to 0.15 in females and 0.14 in males [2]. Furthermore, the occurrence rate of Gd-enhanced lesions, which is an indicator of disease activity, decreased from 54.8% in patients in their 20s to 12.2% after their 50s, further suggesting a decrease in disease activity with age [9]. A previous study has shown that Gd-enhancing lesions in the brain were associated with disease activity and suggested that age correlated more strongly with Gd-enhancing lesions than sex or the use of DMD [3]. Additionally, among patients over 50 years of age, the incidence of T2 and Gd-enhancing lesions decreased, suggesting that disease activity and the contribution of DMD to relapse suppression are reduced in elderly patients with MS. Another previous study analyzed the relapse rate and disability progression between the DMD treatment and untreated groups in elderly patients with MS, and no differences in the relapse rate and disease progression were observed between the two groups [10].

Although the disease activity seemed to decrease after 50 years of age, some patients present disability progression. Recently, SPMS, which is characterized by disability progression, regardless of relapse, has gathered attention. In MS, approximately half of the patients convert to SPMS within 15-20 years of disease onset [1]. The influence on confirmed disability accumulation may be greater by “progression independent of relapse activity (PIRA)” rather than by “relapse-associated disabilities (RAW)” [11]. Additionally, older age at onset, male sex, non-white ethnicity, smoking, high frequency of relapses, short interval between relapses, poor recovery from the first relapse, high EDSS score, and early cognitive impairment are poor prognostic factors in MS [12]. MRI findings associated with a poor prognosis include multiple T2 lesions, Gd-enhancing lesions, lesions in the juxtacortical region, and cerebral atrophy [12]. Moreover, biomarkers such as an increased IgG index, the presence of spinal cord lesions, positive OCB, and elevated levels of neurofilament light chains in the cerebrospinal fluid and serum contribute to poor prognosis. In the present study, older age at the last visit and the presence of brainstem lesions on MRI at 50 years of age were predictors of conversion to SPMS.

In our study, patients in the DMD treatment group had a higher number of poor prognostic factors, such as the presence of spinal cord lesions and increased IgG index, than those in the untreated group (Table 1) [12]. Overall, the OCB positivity rate was low, which may be because the study participants were Japanese and had a lower positivity rate compared to white people, as well as the presence of missing data. The lower positivity rate in the untreated group could be due to the higher proportion of patients with a favorable prognosis. Thus, these results may indicate that DMD treatment tends to be administered to patients with poor prognostic factors. To adjust for treatment selection bias, a logistic regression analysis was performed using propensity scores; no significant differences were observed in relapse, disability progression, or conversion to SPMS, which were the major outcomes of this study. Based on these findings, it was suggested that some elderly patients with MS were stable, no long-term relapse or disease progression occurred in the 13 patients in the untreated group. However, despite a decrease in disease activity with age, some patients experience relapse and/or disability progression; a previous study suggested that the discontinuation of DMD may slightly increase the risk of new lesions on MRI [13].

The pathogenesis of SPMS is unclear, and no effective DMD can suppress the progression of disability until now. However, many recent studies have been conducted on SPMS, for example, patients with SPMS who have meningeal B-cell aggregates show several distinct clinical characteristics, including early disease onset, early age at wheelchair dependence, and more pronounced cortical demyelination [14]. These results indicated that B cells are involved in the pathogenesis of SPMS. In addition, an activated microglia accumulation at the rim of old demyelinated plaques, called “smoldering plaques,” was observed in patients with SPMS; this finding suggests that progressive demyelination occurs even in old demyelinated plaques and is considered one of the causes of the progressive worsening of neurological symptoms in SPMS [15]. In 2018, the inhibitory effects of siponimod, a selective sphingosine 1-phosphate (S1P) receptor 1,5 modulator, on disability progression in SPMS were reported [16]. The identification of risk factors for conversion to SPMS and the early diagnosis of such are important. In this study, we examined the risk factors for the conversion to SPMS after 50 years of age. There were more cases of conversion to SPMS after 50 years of age in patients with brainstem lesions on MRI at 50 years of age than in cases without. This result partially matches previous findings [17]. If brainstem lesions are observed in 50-year-old patients, we believe that the administration or continuation of DMDs, such as siponimod, which is efficacious against SPMS, should be considered.

The limitations of this study include that patients in the DMD treatment group tended to possess poorer prognostic factors, such as the presence of spinal cord lesions and increased IgG index, compared with the untreated group [18]. Although we attempted to exclude confounding factors for treatment selection through logistic regression analysis using propensity scores, we were unable to completely exclude factors related to disease activity regarding treatment selection. Therefore, increasing the number of cases and examining the factors related to disease activity is warranted in future studies. In this study, the treatment group comprises both interferon-beta and new DMDs, and the potential differences in efficacy may suggest that the outcomes could vary when exclusively evaluating new DMDs in the future. Because few patients used highly effective DMDs, a meaningful comparison could not be conducted. Finally, this was a retrospective cohort study, and missing values were observed. Additionally, the majority of the participants were Japanese.

## Conclusions

The present study identified no significant differences in relapse, EDSS progression, and conversion to SPMS between the DMD treatment and untreated groups in elderly patients with MS. Although some patients aged over 50 years exhibited relapse or deterioration, some patients remained without relapse or disability progression, suggesting that the discontinuation of DMD treatment may be considered in some elderly patients without poor prognostic factors. Patients aged 50 years with brainstem lesions have a higher risk of conversion to SPMS compared to patients without such lesions. Therefore, appropriate administration and/or continuation of DMDs that are effective in SPMS, such as siponimod and OMA, should be considered for 50-year-old patients with brainstem lesions.
